# Dental Visits, Dentist–Patient Communication, and Its Association with Oral Cancer Examination Behavior Among Tobacco/Nicotine Product Users in Western India: A Cross-Sectional Study

**DOI:** 10.3390/healthcare14060771

**Published:** 2026-03-19

**Authors:** Nirmal Ahuja, Nikhil A. Ahuja, Dimple Bajaj, Mahima Gulabani, Hitesh Navaparia, Eugene Lengerich, Satish K. Kedia

**Affiliations:** 1 School of Behavioral Sciences and Education, Penn State Harrisburg, Middletown, PA 17057, USA; naa47@psu.edu; 2 Department of Public Health Sciences, Slippery Rock University of Pennsylvania, Slippery Rock, PA 16057, USA; nikhil.ahuja@sru.edu; 3 Department of Public Health Dentistry, Vaidik Dental College and Research Center, Daman 396210, India; dr.dimplebajaj@hotmail.com; 4 Independent Researcher, Grove City, PA 16127, USA; dr.mahi0909@gmail.com; 5 Department of Oral and Maxillofacial Surgery, Vaidik Dental College and Research Center, Daman 396210, India; hiteshnavaparia@gmail.com; 6 Department of Public Health Sciences, Penn State College of Medicine, Hershey, PA 17033, USA; elengerich@pennstatehealth.psu.edu; 7 Division of Social and Behavioral Sciences, School of Public Health, University of Memphis, Memphis, TN 38152, USA

**Keywords:** dental visits, dentist-patient communication, oral cancer, oral cancer examination (OCE), oral cancer screening, tobacco users

## Abstract

**Highlights:**

**What are the main findings?**

- Nearly half (45.9%) of the participants had never visited a dentist, and only 8.7% reported having undergone an oral cancer examination (OCE).
- Those who visited a dentist within the past year were almost 10 times more likely to have had an OCE.
- Dentist–patient communication about the importance of OCEs and the benefits of quitting tobacco was linked to a 2.5-fold increase in OCE behavior.

**What are the implications of the main findings?**

- Promoting regular dental visits among tobacco/nicotine product users is essential, and policymakers should consider mandating oral cancer examinations (OCEs) as part of routine dental care for this high-risk group.
- Strengthening dentist–patient communication about OCEs and tobacco cessation should be a priority for public health programs to help reduce the oral cancer burden among tobacco/nicotine product users.

**Abstract:**

**Background/Objectives:** Regular dental visits are key to early oral cancer detection, especially for tobacco users. Effective dentist–patient communication about oral cancer examinations (OCEs) and quitting tobacco could influence patients’ actual OCE behavior. This study investigated the association of dental visits and dentist–patient communication with OCE behavior among tobacco/nicotine product users in Western India. **Methods:** A cross-sectional study was conducted among 350 current tobacco/nicotine product users from a dental hospital and 15 dental camps in Daman, India, between July 2023 and January 2024. The outcome variable was OCE behavior; the explanatory variables included past dental visits and dentist–patient communication about the benefits of quitting tobacco and importance of OCEs; and the covariates were socio-demographic factors and past 30-day smoking and smokeless tobacco use. The data was analyzed using bivariate and multivariable logistic regression models. **Results:** Only 8.7% of participants reported having undergone an OCE. Those who visited a dentist within the past year were approximately 10 times more likely to have had an OCE (OR = 9.95, *p* = 0.01). Participants whose dentist discussed the importance of OCE (aOR = 2.59, *p* = 0.04) and the benefits of quitting tobacco (OR = 2.45, *p* = 0.04) were 2.5 times more likely to have had an OCE. **Conclusions:** Our findings highlight the important role of dental visits and effective dentist–patient communication in promoting OCEs among tobacco/nicotine product users. Public health campaigns should prioritize enhancing dental visits and dentist–patient communication to emphasize the benefits of tobacco cessation and regular OCEs to reduce the oral cancer burden in this population.

## 1. Introduction

Oral cancer is a highly lethal disease and one of the most debilitating and disfiguring malignancies [1]. Currently, India is the epicenter of oral cancer, accounting for nearly one-third of the global cases [2,3]. India reported 143,759 new oral cancer cases and 79,979 oral cancer deaths in 2022 [4], with incidence rates of 14.7 and 5.0 for men and women, respectively, per 100,000 [4]. While factors like poor oral hygiene, nutritional deficiencies, oral HPV infection, and chronic alcoholism contribute to oral cancer, the primary cause in India is the extensive use of tobacco products, especially smokeless tobacco and areca nut chewing [5]. About 28.6% of Indian adults use tobacco in some form—10.3% smoke, and 21.4% use smokeless tobacco [6]. In the Union Territory of Dadra Nagar Haveli and Daman and Diu in Western India, tobacco use is even more prevalent, with approximately 39% of men and 3% of women consuming tobacco products [7]. Several systematic reviews and meta-analyses have shown that tobacco users face a significantly higher risk of oral cancer and potentially malignant oral disorders [1,8].

Oral cancer examination (OCE) by visual inspection is a simple and cost-effective method for detecting pre-cancerous lesions and early-stage cancer [9]. High-risk individuals, particularly tobacco users, are recommended to undergo OCEs during routine dental or medical visits with a trained provider [10,11]. However, low OCE rates have been reported in the Indian population, ranging from 2% to 16% in different settings [12,13,14]. National Family Health Survey (NFHS-5) data indicates that an abysmal 1.2% of men and 0.9% of women aged 30–49 years in India have ever undergone an OCE [15]. Additionally, 60–80% of oral cancer cases are diagnosed at advanced stages (III or IV), leading to poor 5-year survival rates [11].

Dental practices play a pivotal role in the early detection and prevention of oral cancer, contributing to improved treatment outcomes and survival [16,17]. Routine dental visits provide opportunities for “opportunistic screening,” enabling dentists to perform OCEs during regular appointments [18]. This approach is especially crucial for high-risk populations, such as tobacco users, who often delay seeking medical care until symptoms appear [19]. Evidence suggests that more frequent dental visits are associated with earlier diagnosis of oral and pharyngeal cancers [20]. A systematic review and meta-analysis further found that individuals who infrequently or irregularly visit the dentist are more likely to develop head and neck cancers [21].

Another effective strategy for improving the early diagnosis and prevention of oral cancer is to enhance dentist–patient communication about the health benefits of quitting tobacco and the importance of OCEs. Effective communication empowers patients with the knowledge needed to make informed decisions about their own oral health [22]. However, previous research indicates poor dentist–patient communication related to oral cancer [23]. A national UK survey of 3384 adults found that only 7.1% had been informed by their dentist or doctor about oral cancer [24]. Similarly, in a cross-sectional study of 184 dental patients, only 14% knew they had been screened and just 12% reported ever receiving screening [25]. Studies from the UK, US, and other high-income countries indicate that dentists often perform OCEs without informing patients and may avoid using the term “cancer” due to concerns about causing alarm or perceived patient resistance [25,26,27]. In contrast, research also shows that dental patients, including those at risk of developing oral cancer, are in favor of discussing the disease with their dentists [27]. Another survey indicated that 92% of patients wanted to be informed when screened, and 97% desired support in reducing their oral cancer risk [25].

Although prior research highlights the role of dental visits and dentist–patient communication in early oral cancer detection, limited evidence exists on how these factors influence OCE behavior among high-risk groups, such as tobacco users, in India. Most existing studies have been conducted in Western countries, where oral cancer incidence is lower, smokeless tobacco use is less prevalent, routine preventive dental visits are more common, and patient-centered communication practices are more systematically integrated into care [19,23,26]. In contrast, India faces a higher oral cancer burden, widespread use of smokeless tobacco products, variable access to dental services, and sociocultural norms that may influence health communication and care-seeking behavior [3,5]. This study aims to examine the associations of past dental visits and dentist–patient communication with OCE behavior among tobacco/nicotine product users in Western India. The findings will help inform targeted interventions and policies to increase OCE rates in this high-risk population, ultimately improving early detection and reducing the burden of oral cancer.

## 2. Materials and Methods

### 2.1. Study Design, Study Setting, and Study Population

This cross-sectional study was conducted in Daman, a city in the Indian Union Territory of “Daman and Diu” in Western India. Participants were recruited from a dental hospital and 15 dental camps conducted by the dental hospital at several locations in Daman between July 2023 and January 2024. The inclusion criteria to participate in this study were: (1) aged 18 years and above; (2) a current (past 30 days) user of smoking tobacco (cigarettes, bidis, hookah), electronic cigarettes (e-cigarettes), smokeless tobacco (local products like kharra, khaini, Gutkha, mishri, paan masala with tobacco, paan with tobacco, zarda, chewing tobacco, snuff), or any other combination of these products; and (3) the ability to understand the consent procedure. We excluded individuals who had: (1) a history of oral cancer or were referred for assessment of suspicious oral mucosal/pre-malignant lesions or (2) severe debilitating conditions (e.g., neurological disorders such as dementia or Alzheimer’s disease) that could limit their ability to understand the questionnaire and participate in the study.

Sample size was determined using a priori power analysis based on the expected proportion of participants undergoing OCEs. Previous studies have reported approximately 2–16% OCE rates among the general adult population in India [13,14]. Given that our sample comprised tobacco users who are at increased risk for oral cancer, we conservatively assumed a 2% OCE rate [28]. With a significance level of 0.05, 80% power, and an anticipated 20% non-response rate, the required sample size was estimated at 350 participants.

### 2.2. Data Collection Procedure

The data for this study was collected by a team of 14 dental interns working at a dental hospital. These data collectors were trained by two study investigators (NA and NA) and were supervised in the data collection process by another investigator (DB) at the study site. Study participants were screened for eligibility based on the inclusion and exclusion criteria by one of our investigators (DB) and the data collection team. All eligible participants who agreed to participate were interviewed in a private setting by the data collection team using the study measures (see below for details). The questionnaire was adapted from previous studies and translated into Hindi, a locally spoken language in the study area, using a forward–backward translation process to ensure language validity. Prior to the start of the study, the questionnaire was pilot-tested with 15 participants to assess its face validity, clarity, comprehension, response interpretation, and feasibility. The participants reported that the questions were generally clear and understandable. Minor wording modifications were made to simplify terminology, improve the clarity of certain items, and enhance the flow of the questionnaire. No major structural changes were required.

### 2.3. Measures

#### 2.3.1. Outcome Variable

*OCE Behavior:* The OCE behavior of the participants was assessed using two questions adapted from previous studies [25,29,30]. These two items have demonstrated acceptable reliability (r = 0.79) [25,29,30]. Since dentists or health professionals might conduct OCEs without notifying patients, some individuals who have undergone an examination may not recognize it. Therefore, each participant was asked two questions about their OCE behavior. The first question asked whether the participant had ever had an OCE, *"Have you ever had an oral cancer examination?"*, with response options of “Yes”, “No”, or “Don’t know”. If the answer was “No” or “Don’t know”, participants were given a description of the OCE procedure and asked a second question, *"Has a doctor, dentist, or any other health professional ever examined your mouth by pulling on your tongue and feeling under your tongue and inside your cheeks?"*, with response options of “Yes”, “No”, or “Don’t know”. Participants who answered “Yes” to either question were categorized as having ever had an OCE, while those who answered “No” or “Don’t know” were categorized as not having had one.

#### 2.3.2. Explanatory Variables

*Past Dental Visit:* Participants’ history of past dental visits was assessed using a single item: *“How long ago was your last visit to the dentist?”*. The response options were “I have never visited the dentist”, “within the past 12 months”, “1 to 2 years ago”, and “more than 2 years ago”.

*Dentist–Patient Communication:* Two items were used to assess dentist–patient communication regarding the benefits of quitting tobacco and the importance of OCEs. The first question asked, “*Did the dentist have a direct conversation with you about the health benefits of giving up cigarettes and other tobacco products?*” The second question asked, *“Did the dentist have a direct conversation with you about the importance of examining your mouth for oral cancer?”* The response options for both questions were “Yes”, “No”, and “Don’t know”. These questions were adapted from National Health and Nutrition Examination Survey (NHANES), a nationally representative survey conducted by the US Centers for Disease Control and Prevention. NHANES instruments undergo extensive cognitive testing, pilot testing, standardized interviewer training, and ongoing quality control procedures to ensure reliability and validity and have been utilized in prior studies [30,31,32].

#### 2.3.3. Covariates

Participants’ demographic and tobacco use characteristics were considered as covariates in this study. These included age, sex (male, female), marital status (single/never married, married), socio-economic status (SES) measured using the Modified Kuppuswamy SES scale (upper class/upper-middle, lower-middle, upper-lower, lower) [33], number of family members (one, two to four, four or more), and number of minor children (none, one, two or more). Participants’ tobacco/nicotine product use was categorized as smoking tobacco use, e-cigarette use, smokeless tobacco use, or dual use (yes/no for each). Those who reported using cigarettes, bidis, or hookah were classified as smoking tobacco users, while those who reported using electronic cigarettes were classified as e-cigarette users. Participants who reported using kharra, khaini, gutkha, mishri, paan masala with tobacco, paan with tobacco, zarda, chewing tobacco, or snuff were classified as smokeless tobacco users. Individuals who reported using more than one type of product (i.e., both smoking and smokeless tobacco) were categorized as dual users.

### 2.4. Data Analysis

Descriptive statistics are reported as frequencies and percentages for categorical variables and as means and standard deviations for continuous variables. Bivariate and multivariable logistic regression analyses were conducted to examine associations of dental visits and dentist–patient communication with OCEs. Odds ratios (ORs) from the bivariate analysis and adjusted odds ratios (aORs) from the multivariable analysis, along with their 95% confidence intervals (CIs) and significance levels, are reported. We calculated the “Firth bias-corrected estimates” of the OR and 95% CI to address potential sparse data bias or separation issues for the sex variable. Statistical analyses were performed using SAS 9.4, with the significance level set at *p* ≤ 0.05.

## 3. Results

The mean age of the participants was 36.1 years (SD ± 11.5). Many of the participants were male (98.8%), married (80.4%), and belonged to the upper-lower socio-economic class (52.3%). Over half (58.3%) of the participants lived in households with more than four family members, and 38.6% had two or more minor children in the house. Smokeless tobacco use was highly prevalent (88%) among the participants, along with 22.3% smoking cigarettes and 12% both smoking and using smokeless forms of tobacco (Table 1). None of the participants reported using e-cigarettes. Nearly half of the participants (45.9%) had never visited a dentist; 13.7% had visited within the past 12 months; 10.2% had visited 1–2 years ago; and 30.2% had visited more than 2 years ago. Of those who had a past dental visit, only 37.2% indicated that their dentist discussed the health benefits of quitting cigarettes and other tobacco products, and only 28.7% reported their dentist discussing the importance of OCEs. The prevalence of having ever undergone an OCE was a meager 8.7% among the study participants (Table 1).

Bivariate logistic regression analysis identified several factors associated with participants having ever received an OCE (Table 2). With each additional year increase in the participant’s age, the likelihood of having had an OCE increased by 4% (OR = 1.04, 95% CI: 1.01–1.07, *p* = 0.02). Participants of upper/upper-middle SES were approximately 4.5 times more likely to have had an OCE (OR = 4.47, 95% CI: 1.53–37.79, *p* = 0.006) compared to those of lower SES. Living in households with more than four family members decreased the likelihood of participants to have had an OCE by 76% (OR = 0.24, 95% CI: 0.05–0.98, *p* = 0.03) compared to living with only one family member. Participants who had visited a dentist within the past year were approximately 10 times more likely to have had an OCE (OR = 9.95, 95% CI: 2.89–34.29, *p* = 0.01) compared to those who had never visited a dentist. Additionally, those who indicated their dentist discussing the health benefits of quitting cigarettes and other tobacco products were approximately 2.5 times more likely to have had an OCE (OR = 2.45, 95% CI: 1.04–5.78, *p* = 0.04) than those who reported not having discussed this information. Similarly, participants who reported that their dentist discussed the importance of OCEs were approximately 2.7 times more likely to have had an OCE (OR = 2.65, 95% CI: 1.11–6.30, *p* = 0.03) than those who reported not having discussed this information.

Results from the multivariable logistic regression analysis are shown in Table 3. Compared to those who had never visited a dentist, participants who had visited the dentist within the past one to two years were approximately 10 times more likely to have had an OCE (aOR = 10.33, 95% CI: 2.45–43.54, *p* = 0.04). Additionally, participants who reported that their dentist discussed the importance of OCEs were roughly 2.6 times more likely to have had an OCE (aOR = 2.59, 95% CI: 1.01–6.67, *p* = 0.04) than those who reported not having discussed this information.

## 4. Discussion

This study offers valuable insights into the influence of dental visits and dentist–patient communication on tobacco/nicotine product users’ OCE behavior in Western India. Nearly half (45.9%) of the participants had never visited a dentist, and only 8.7% reported having undergone an OCE in this study. This is concerning given the high prevalence of tobacco use in India and its strong association with oral cancer [5,34]. Previous studies have similarly reported lower rates of dental visits [35,36] and OCE [37,38,39] among tobacco users, underscoring the urgent need for increased awareness and targeted interventions aimed at promoting routine dental care and OCEs. Our findings further validate that both dental visits and effective dentist–patient communication significantly influence OCEs in this population.

One of the most notable findings from our study is the strong association between participants’ past dental visits and OCE uptake. Those who had visited a dentist within the past year or 1–2 years were about 10 times more likely to have had an OCE. These results underscore the critical role that dental visits play in OCE uptake among these patients. Evidence shows that regular dental visits, including visits within the past 12 months, increase the likelihood of receiving an OCE and contribute to early diagnosis of oral cancer [20,21,40,41]. However, the lower rate of dental visits reported in this study suggests that many tobacco users are missing these crucial opportunities for timely screening. Improving access to and utilization of dental services may therefore be essential for increasing OCE uptake. Strategies such as expanding dental coverage, emphasizing ethical obligations for dentists to conduct OCEs, offering financial incentives or subsidies, strengthening community outreach, and integrating OCEs into routine dental care policies could enhance early detection of oral cancer [20,21,42]. Mandating or formally incorporating OCEs into standard dental visits and national oral health policies, particularly for tobacco users, may further institutionalize screening practices and reduce late-stage oral cancer diagnoses.

Dentist–patient communication emerged as another key factor influencing OCEs in this study. Participants whose dentists discussed the health benefits of quitting tobacco and the importance of OCEs were nearly two and a half times more likely to have undergone an OCE. This finding aligns with those of previous studies demonstrating that effective communication promotes preventive behaviors and increases acceptance of recommended screenings [22,42,43]. Importantly, evidence suggests that dental patients, particularly those at high risk of oral cancer, generally welcome discussions about tobacco cessation and OCEs and prefer to be informed when screened [23,25,42,44]. Despite this, communication gaps remain substantial. In our study, only 37.2% of participants reported discussions about tobacco cessation, and less than one-third recalled conversations about the importance of OCEs with their dentist. Similar findings have been observed in previous studies, where dentists often avoid explicitly discussing oral cancer or fail to inform patients when screening is performed [23,26,27,45]. The contributing factors may include limited dental provider knowledge, uncertainty about screening procedures, and concerns about causing patient anxiety [23,26,27,45]. However, research indicates that evidence-based communication to discuss oral cancer does not increase patient distress [45]. Strengthening dental provider training, improving their knowledge of risk factors and screening protocols, and implementing standardized communication guidelines may therefore enhance both the frequency and quality of oral cancer discussions in dental settings [46].

In our study, we found that each additional year of age increased the likelihood of having undergone an OCE by 4%. This highlights how age may play a role in preventive health behaviors like undergoing OCEs. Older individuals may experience increased oral health symptoms that prompt dental visits, leading to increased OCEs [47,48]. Furthermore, as individuals age, especially tobacco users, their risk of oral cancer increases due to decreased resilience and increased permeability of the oral mucosa, which may compel them to undergo an OCE [48]. Household composition also emerged as a significant factor influencing OCEs in our study. Participants living in households with more than four family members were 76% less likely to have had an OCE compared to those living with only one family member. This finding suggests that in larger households, competing demands on time and financial resources may make it difficult to prioritize healthcare, limiting individuals’ ability to seek preventive services like OCEs and affecting their oral health-related quality of life [49,50]. Public health initiatives should consider household dynamics and potential resource constraints when designing programs to promote awareness about early diagnosis of oral cancer.

Socio-economic status (SES) also significantly influenced participants’ likelihood of receiving an OCE in the current study. Those from upper or upper-middle SES backgrounds were nearly 4.5 times more likely to have had an OCE compared to those from lower SES backgrounds (OR = 4.47). This finding aligns with the existing literature that highlights disparities in oral cancer screening participation based on socio-economic level, with those of higher SES more likely to be aware of and screened for oral cancer [51,52,53]. Barriers such as cost, lack of insurance, and limited access to healthcare resources may hinder lower-SES individuals from seeking dental care and, consequently, receiving an OCE [54]. This is problematic because oral cancers are more prevalent in low-SES groups [55]. Future oral cancer awareness and screening campaigns should be directed at vulnerable and low-SES groups.

This study has several limitations that should be acknowledged. First, the cross-sectional design restricts the ability to infer causality between the variables studied and limits the ability to establish temporal relationships between dental visits, dentist–patient communication, and OCE behavior. Longitudinal studies are needed to clarify the directionality of these associations. Second, the reliance on self-reported data may have introduced recall bias or social desirability bias. Participants may not accurately recall prior OCEs or discussions with their dentist, and objective verification through dental records was not possible. Additionally, several key variables, including OCE behavior and dentist–patient communication, were assessed using dichotomous (yes/no) response options. While this approach facilitates clarity and ease of administration, it limits the ability to capture the frequency, quality, depth, or contextual nuances of these interactions and may oversimplify complex behaviors. Future studies should consider using scaled or multi-item measures to provide more detailed assessments. Third, the sample was predominantly male (98.8%), limiting generalizability across genders, as most women were unable to participate due to time constraints and other commitments. Since patterns of dental visits, dentist–patient communication, and OCE behavior may differ among female and other gender identities, future studies should focus on exploring these gender differences. Fourth, while the study adjusted for some covariates, other factors that could also influence OCEs, such as risk perceptions, health literacy, and access to dental care, were not included in the analysis. Fifth, the study did not collect information regarding the duration of smoking or tobacco use. The absence of this measure limits our ability to assess dose–response relationships or examine the impact of cumulative tobacco exposure on OCE behavior. Future studies should incorporate measures of duration and intensity of tobacco use to provide a more comprehensive understanding of OCE behaviors among tobacco users. Sixth, the study did not assess the quality or depth of the communication between dentists and patients. While participants reported whether their dentist discussed the benefits of quitting tobacco and importance of OCEs, the quality of these conversations (e.g., clarity, patient understanding, or persuasiveness) was not evaluated. Future research should employ qualitative or mixed-methods designs to gain a deeper understanding of the contextual and experiential aspects of dentist–patient communication and OCE among tobacco users.

Potential limitations regarding the study’s participant sample should also be acknowledged. Participants were recruited exclusively from a dental hospital and dental camps, which may have introduced selection bias. Individuals who seek care in these dental settings may differ from the broader population of tobacco/nicotine product users in terms of their health-seeking behavior, oral health awareness, or access to dental services. As a result, the findings may not be fully generalizable to the wider population of tobacco/nicotine users. Further, only 29 participants (8.3%) reported ever undergoing an OCE, resulting in fewer events per variable than recommended for multivariable logistic regression. This limited the ability to detect precise associations between predictors and OCE behavior. Future studies with larger numbers of participants who have undergone an OCE should explore these associations more robustly. Finally, caution should be taken when extrapolating these findings to broader or more diverse populations and regions, as the study was conducted among tobacco users in Western India. Cultural, socio-economic, and healthcare access differences in other regions of India or other countries may lead to differing patterns of dental visits, dentist–patient communication, and OCE behavior.

## 5. Conclusions

Our findings highlight the significant role of both past dental visits and effective dentist–patient communication in influencing OCE behavior among tobacco/nicotine product users in Western India. Public health campaigns should prioritize improving dentist–patient communication and encouraging regular dental visits, highlighting the benefits of tobacco cessation and routine OCEs, to help reduce the oral cancer burden in this population.

## Figures and Tables

**Table 1:** Descriptive statistics of participant characteristics.

Characteristics	*n* (%)
Age (Mean ± SD) (*n* = 343)	36.1 ± 11.5
Sex (*n* = 343)	
Male	339 (98.8)
Female	4 (1.2)
Socio-economic status (SES) (*n* = 350)	
Upper/Upper-middle class	51 (14.6)
Lower-middle class	98 (28.0)
Upper-lower class	183 (52.3)
Lower class	18 (5.1)
Marital status (*n* = 347)	
Single, never married	68 (19.6)
Married	279 (80.4)
Number of family members in the house (*n* = 343)	
1	13 (3.8)
2–4	130 (37.9)
4+	200 (58.3)
Number of minor children in the house (*n* = 345)	
None	114 (33.0)
1	98 (28.4)
2 or more	133 (38.6)
Smoking tobacco use (*n* = 350)	
Yes	78 (22.3)
No	272 (77.7)
Smokeless tobacco use (*n* = 350)	
Yes	308 (88.0)
No	42 (12.0)
Dual tobacco use (smoking + smokeless) (*n* = 343)	
Yes	42 (12.0)
No	301 (88.0)
Past dental visit (*n* = 344)	
Never visited dentist	158 (45.9)
Within the past 12 months	47 (13.7)
1–2 years	35 (10.2)
More than 2 years	104 (30.2)
Dentist conversation about the benefits of giving up cigarettes and other tobacco products (*n* = 183) ^a^	
Yes	68 (37.2)
No	115 (62.8)
Dentist conversation about the importance of examining your mouth for oral cancer (*n* = 181) ^a^	
Yes	52 (28.7)
No	129 (71.3)
Ever had oral cancer examination (*n* = 332)	
Yes	29 (8.7)
No	303 (91.3)

SD = standard deviation; percentages are based on the number of respondents for each variable. Missing data was excluded from the counts; ^a^ denotes questions asked only of respondents who reported a past dental visit.

**Table 2:** Bivariate logistic regression analysis examining association of demographic characteristics, past dental visit and dentist–patient communication with oral cancer examination behavior.

Characteristics	Ever Had Oral Cancer Examination	
	OR (95% CI)	*p*-Value
Age	**1.04 (1.01–1.07)**	**0.02**
Sex ^a^		
Male	Ref	
Female	1.11 (0.04–29.84)	0.94
Socio-economic status (SES)		
Upper/Upper-middle class	**4.47 (1.53–37.79)**	**0.006**
Lower-middle class	0.81 (0.09–7.69)	0.19
Upper-lower class	1.45 (0.18–11.73)	0.91
Lower class	Ref	
Marital status		
Single, never married	Ref	
Married	1.22 (0.45–3.33)	0.69
Number of family members in the house		
1	Ref	
2–4	0.41 (0.09–1.66)	0.67
4+	**0.24 (0.05–0.98)**	**0.03**
Number of minor children in the house		
None	Ref	
1	0.52 (0.20–1.36)	0.59
2 or more	0.44 (0.18–1.09)	0.26
Smoking tobacco use		
Yes	0.51 (0.17–1.52)	0.23
No	Ref	
Smokeless tobacco use		
Yes	1.82 (0.42–7.98)	0.43
No	Ref	
Dual tobacco use (smoking + smokeless)		
Yes	0.50 (0.12–2.19)	0.36
No	Ref	
Past dental visit		
Never visited dentist	Ref	
Within the past 12 months	**9.95 (2.89–34.29)**	**0.01**
1–2 years	7.82 (0.37–29.52)	0.10
More than 2 years ago	4.01 (0.52–13.17)	0.88
Dentist conversation about the benefits of giving up cigarettes and other tobacco products		
Yes	**2.45 (1.04–5.78)**	**0.04**
No	Ref	
Dentist conversation about the importance of examining mouth for oral cancer		
Yes	**2.65 (1.11–6.30)**	**0.03**
No	Ref	

OR, odds ratio; CI, confidence interval; Ref, reference; ^a^ Firth’s penalized likelihood approach was used to reduce bias in the parameter estimates; bold values denote statistically significant results at *p* ≤ 0.05.

**Table 3:** Multivariable logistic regression analysis examining association of past dental visit and dentist–patient communication with oral cancer examination behavior.

Characteristics	Ever Had Oral Cancer Examination	
	aOR (95% CI)	*p*-Value
Past dental visit		
Never visited dentist	Ref	
Within the past 12 months	8.08 (0.89–29.89)	0.08
1–2 years	**10.33 (2.45–43.54)**	**0.04**
More than 2 years	4.04 (0.19–13.58)	0.84
Dentist conversation about the benefits of giving up cigarettes and other tobacco products		
Yes	1.76 (0.69–4.50)	0.24
No	Ref	
Dentist conversation about the importance of examining mouth for oral cancer		
Yes	**2.59 (1.01–6.67)**	**0.04**
No	Ref	

aOR, adjusted odds ratio; CI, confidence interval; Ref, reference; analysis adjusted for age, SES, marital status, number of family members, number of minor children, smoking and smokeless tobacco use; bold values denote statistically significant results at *p* ≤ 0.05.

## Data Availability

The raw data supporting the conclusions of this article will be made available by the authors on request due to ethical reasons.

## Abbreviations

The following abbreviations are used in this manuscript:

OCE	Oral cancer examination
GLOBOCAN	Global Cancer Observatory
SES	Socio-economic status
OR	Odds ratio
aOR	Adjusted odds ratio
CI	Confidence interval
PASSHE	Pennsylvania State System of Higher Education
